# Differential Variance Analysis: a direct method to quantify and visualize dynamic heterogeneities

**DOI:** 10.1038/srep43496

**Published:** 2017-03-14

**Authors:** Raffaele Pastore, Giuseppe Pesce, Marco Caggioni

**Affiliations:** 1 CNR–SPIN, sezione di Napoli, Via Cintia, 80126 Napoli, Italy; 2 University of Cincinnati, UC Simulation Center, 2728 Vine Street, Cincinnati, OH 45219, USA; 3 Corporate Engineering, The Procter & Gamble Company, Cincinnati, 8256 Union Centre Blvd., West Chester, OH 45069, USA; 4 Università di Napoli “Federico II”, Dipartimento di Fisica, Via Cintia, 80126 Napoli, Italy

## Abstract

Many amorphous materials show spatially heterogenous dynamics, as different regions of the same system relax at different rates. Such a signature, known as Dynamic Heterogeneity, has been crucial to understand the nature of the jamming transition in simple model systems and is currently considered very promising to characterize more complex fluids of industrial and biological relevance. Unfortunately, measurements of dynamic heterogeneities typically require sophisticated experimental set-ups and are performed by few specialized groups. It is now possible to quantitatively characterize the relaxation process and the emergence of dynamic heterogeneities using a straightforward method, here validated on video microscopy data of hard-sphere colloidal glasses. We call this method Differential Variance Analysis (DVA), since it focuses on the variance of the differential frames, obtained subtracting images at different time-lags. Moreover, direct visualization of dynamic heterogeneities naturally appears in the differential frames, when the time-lag is set to the one corresponding to the maximum dynamic susceptibility. This approach opens the way to effectively characterize and tailor a wide variety of soft materials, from complex formulated products to biological tissues.

Many complex fluids, when changing control parameters like temperature or composition, exhibit a jamming transition from a liquid-like to an amorphous solid-like state. Approaching such a transition, the dynamics dramatically slows down and shows increasing spatio-temporal fluctuations, known as Dynamic Heterogeneities (DHs)^1^. This dynamic signature is especially relevant for glass forming systems, such as supercooled liquids and dense colloidal suspensions: since the glass transition has been not yet related to a clear structural variation^2^, its fingerprint remains essentially of a dynamic type, hidden in the way the system moves. Indeed, in liquids close to the glass transition, DHs emerge as transient clusters of particles with a mobility larger or smaller than the average^3^. The size and the lifetime of these dynamical clusters increase on approaching the transition, playing a role similar to density fluctuations close to an ordinary critical point^4^,^5^,^6^. This motivated the glass community to develop a robust framework for characterizing DHs. In glass forming liquids, the structural relaxation process as a function of time, Δ*t*, can be monitored through a dynamic order parameter probing the local motion on the length scale of the particle size. Different experimentally measured probes, such as the dynamic scattering functions or the persistence, are good choice as order parameter and provide similar information^7^. The fluctuations of the dynamic order parameter define a dynamic susceptibility, *χ*_4_(Δ*t*), that allows for quantifying the degree of DH. Alternatively, *χ*_4_(Δ*t*) is also defined as the space integral of a correlation function, *G*_4_(*r*, Δ*t*), measuring the correlation of the displacements over Δ*t* between particles separated by a distance *r*. These equivalent definitions of *χ*_4_ reveal the two faces of DHs, that can be viewed either as ensemble fluctuations of the dynamic order parameter, or as spatial correlations in the displacement field^1^,^8^.

While direct evidences of DHs have been first provided by numerical simulations^9^,^10^, their existence have been directly confirmed by experiments on colloidal glasses and other colloidal systems, such as gels and foams^11^,^12^,^13^,^14^ and, recently, even in epithelial cell tissues^15^,^16^,^17^.

In fact, DH characterization still remains a complex experimental task, typically handled by a limited number of specialized academic groups, since it requires to resolve the dynamics in space and time, and estimate deviations from the average behavior. As far as individual particle can be resolved, optical or confocal microscopy, combined with particle tracking allows for properly monitoring the macroscopic dynamics, measuring the dynamic order parameter as well as other complex dynamic correlation functions, such as the bond-orientational correlation function^18^. This route is also fundamental to obtain indirect and visual insights on DHs in colloidal systems^3^. Particle tracking based quantification of DHs has been, instead, mainly performed in granular systems of large, non-thermal particles^19^,^20^,^21^. In analogy with numerical simulations^22^, a dynamic susceptibility, *χ*_4_(*l*, Δ*t*), is measured from the fluctuations of the fraction of particles which moved more than an arbitrary chosen cutoff distance, *l*, over the time, Δ*t*^19^,^20^. Alternatively, the cutoff distance can be determined uniquely by the topology, for example by considering the fraction of sample which remains inside the same Voronoi cell across Δ*t*^21^. However, these measurements are difficult and not always possible, especially in crowded colloids, since the length and the overall number of trajectories are often limited, even when particles are clearly resolved. Moreover, particle tracking relies on quite complex algorithms and suffers possible biases due to users’ choice of tracking parameters. By contrast, Dynamic Light Scattering (DLS) based techniques are probably the most robust approach to measure *χ*_4_^23^,^24^, but do not provide any direct visualization of DHs. Simultaneous visualization and quantitative measurement of DHs have been obtained using more sophisticated techniques, such as the Photon Correlation Imaging (PCI), that combines features of both dynamic light scattering and imaging^25^. Results on flowing systems suggest that *χ*_4_ can be measured using simpler methods, based on autocorrelation of image intensity^26^,^27^, and call for further exploring this direction. Recently, elegant approaches to investigate soft matter dynamics analyzing image difference (the same signal exploited in this work) have been developed^28^,^29^. However, the current differential methods do not allow DH characterization. For instance, Differential Dynamic Microscopic (DDM), that provides information similar to DLS from video microscopy data, is an easy and promising technique^30^,^31^, but currently limited to monitor the structural relaxation^32^,^33^ and not DHs. It appears clearly that an easy way to characterize complex fluids with dynamic heterogeneity is highly desirable, also considering that soft glassy materials are common in technological applications and biological systems. In this paper, we introduce a novel and straightforward experimental method to fully characterize DHs in colloidal suspensions and apply it to a popular model system of hard-sphere colloidal glass^34^,^35^,^36^,^37^,^38^ imaged by optical microscopy. Our method utilizes as primary signal the *differential frames* obtained by subtracting images taken at different time. This is also the signal used by DDM, before performing a Fourier analysis and accessing the intermediate scattering function by appropriate fitting of the image structure function^30^. Our Differential Variance Analysis (DVA), instead, does not require Fourier analysis or fitting ansatzs. Indeed, we simply focus on the real space variance of differential frames and its fluctuations to obtain the dynamic order parameter and the dynamic susceptibility, respectively. We validate the result of DVA by performing established single particle tracking analysis and demonstrating that the dynamic order parameter obtained from DVA closely matches the commonly measured Intermediate Self Scattering Function (ISSF) at a wave-length of the order of the particle size. In addition, the differential frames provide a very direct visualization of DHs: the framework we introduce to this aim allows for visualizing DHs not only as spatial correlations, but also as ensemble fluctuations. The key of this visualization is to consider differential frames close to the time-lag Δt^*^, which is determined by the dynamics and can be easily measured from DVA as the time corresponding to the maximum of *χ*_4_.

## Results

### Differential Variance Analysis of glassy dynamics

**Figure 1:**
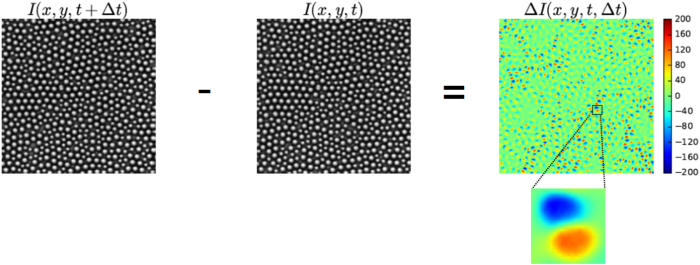
Differential frame. From left to right, two snapshots of a portion of a sample separated by a time-lag Δ*t* = 10 *s*, and the resulting differential frame. Particles that move significantly during the interval Δ*t* give rise in the differential frames to coupled spots of high and low intensity, which look like dipoles, as highlighted by the zoom. The volume fraction of this sample is Φ = 0.71.

**Figure 2:**
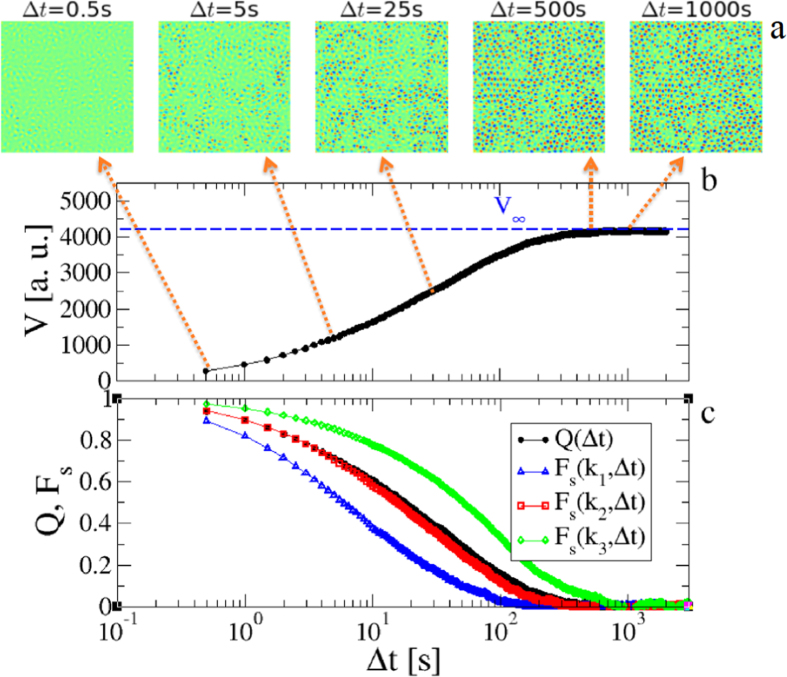
From differential frames to the dynamic order parameter. (**a**) Sequence of differential frames at different time-lags, Δ*t*, as indicated. (**b**) Average intensity variance of differential frames, *V*, as a function of the time-lag Δ*t. V*(Δ*t*) increases up to reach a plateau *V*_∞_. (**b**) The DVA dynamic order parameter, *Q*(Δ*t*), is compared to the ISSF, *F*_*s*_(*k, t*), computed from single particle trajectories, for three wavectors *k*_1_ = 2.3 *μm*^−1^, *k*_2_ = 1.7 *μm*^−1^ and *k*_3_ = 1.15 *μm*^−1^, as indicated. The volume fraction of the sample under consideration is Φ = 0.71.

Data are obtained from a previous experiment^39^ that investigated a quasi two-dimensional mixture of micron-sized hard-sphere-like beads in water (see Methods). In this popular model system of colloidal glass, the dynamics slows down on increasing the colloidal volume fraction. From optical video microscopy of these samples, we consider two frames at time *t* and *t* + Δ*t*, and the *differential frame* generated by the differences between their pixel intensities, Δ*I*(*x, y, t*, Δ*t*) = *I*(*x, y, t* + Δ*t*) − *I*(*x, y, t*), as illustrated in Fig. 1 for two frames of a sample at Φ = 0.71 and separated by a time-lag Δ*t* = 10 *s*, somewhat smaller than the structural relaxation time. On this timescale, some particles move over a distance comparable to their size, while other particles stay localized close to their initial position^3^. Such a scenario clearly emerges from the differential frame. Indeed, a color scale for the differential intensity signal highlights the presence of patterns formed by two adjacent spots of negative and positive Δ*I*, that appear blue and red, respectively. These spots arise as a consequence of detectable single particle movements: a blue spot corresponds to groups of pixels which are occupied by a particle at time *t* but not at time *t* + Δ*t*, and vice-versa for a red spot. Thus, each pair of blue and red spot can be viewed as a dipole or as an arrow representing the particle displacement. By contrast, the green background corresponds to regions occupied by particles that at time *t* + Δ*t* are still localized in their original position (at time *t*), with thermal rattling around this position resulting in small deviations from Δ*I* = 0. To qualitatively illustrate the system temporal evolution, Fig. 2a shows a sequence of these differential frames at increasing time-lags. Initially, as Δ*t* increases, more and more particles move, leaving dipoles in the differential frames. At time-lags much larger than the relaxation time, instead, all particles have moved far away from from their original position and the number of dipoles seems to saturate. Quantitatively, this temporal evolution can be captured by the variance of Δ*I* over pixels (*x, y*):


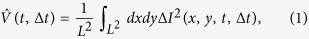


where *L* is the size of the image. Figure 2b shows 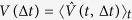, obtained by averaging 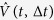 over an ensemble of differential frames with different initial times *t*. A comparison with Fig. 2a, which refers to the same volume fraction, Φ = 0.71, clarifies that in the time window in which the number of dipoles increases, *V*(Δ*t*) also increases, whereas it approaches a plateau, *V*_∞_, at long time, when the number of dipoles saturates. This suggests that the behaviour of the variance is closely related to the relaxation process. Indeed, we are going to show that the average and the fluctuations of 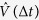 can be used to quantitatively describe the structural relaxation and the emergence of dynamic heterogeneities, respectively. To this aim, we introduce the function


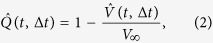


and speculate that its average, 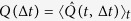, properly describes the structural relaxation. To demonstrate this point, we measure the commonly used ISSF, *F*_*s*_(*k*, Δ*t*) = 〈Ψ_*t*_(*k*, Δ*t*)〉_*t*_, where 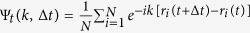, *N* is the number of particles under consideration, and the trajectories *r*_*i*_(*t*) are obtained trough single particle tracking^40^,^41^. Figure 2c shows *Q*(Δ*t*), obtained from the variance in panel a, and *F*_*s*_(*k*, Δ*t*) for three values of the wave-vector, *k* = 2*π*/*λ*, selected in a range relevant to describe the structural relaxation: *k*_1_, *k*_2_ and *k*_3_, corresponding to wave-lengths *λ*_1_ = *d, λ*_2_ = 1.35*d*, and *λ*_3_ = 2*d*, respectively, with *d* ≃ 2.7 *μm* being the average particle diameter. Strikingly, *Q*(Δ*t*) lies between *F*_*s*_(*k*_1_, Δ*t*) and *F*_*s*_(*k*_3_, Δ*t*) and nearly overlaps to *F*_*s*_(*k*_2_, Δ*t*) at any time^42^.

**Figure 3:**
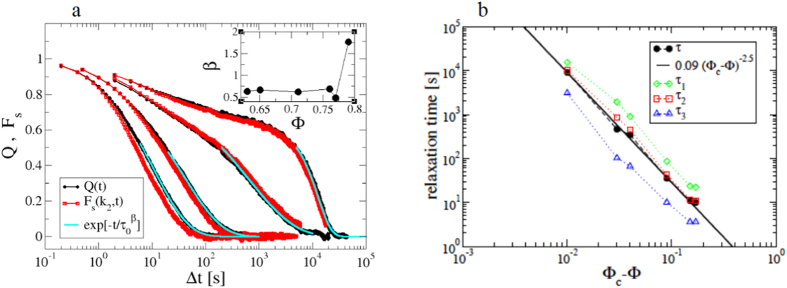
Structural relaxation. (**a**) DVA dynamic order parameter, *Q*(Δ*t*), and ISSF, *F*_*s*_(*k*_2_, Δ*t*), with *k*_2_ = 1.7 *μm*^−1^ at volume fractions Φ = 0.65, 0.71, 0.77, 0.79, from right to left. *Q*(Δ*t*) is nearly overlapped to *F*_*s*_(*k*_2_, Δ*t*) over the whole range of time and investigated volume fractions. The solid lines are fits, 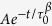, to the late decay of *Q*(Δ*t*). The resulting exponent *β* as a function of Φ is reported in the Inset. (**b**) Relaxation time measured from the *Q* decay, *τ*, and from the ISSF decays, *τ*_1_, *τ*_2_, *τ*_3_ (corresponding to wave-vectors *k*_1_, *k*_2_ and *k*_3_, respectively), as a function of Φ_*c* _− Φ, with Φ_*c*_ = 0.8. *τ* is well fitted by a power-law (Φ_*c* _− Φ)^−2.5^ (solid line). The ISSF relaxation times follow a similar behaviour, with *τ*_2_ being nearly overlapped to *τ*.

In addition, Fig. 3a shows that the close similarity between *Q*(Δ*t*) and *F*_*s*_(*k*_2_, Δ*t*) is manifested over the whole range of investigated volume fractions. Overall, these results clarify that *Q*(Δ*t*) is an effective dynamic order parameter of the system structural relaxation, probing a length scale of the order of one particle diameter. This length scale is not arbitrary, but self-determined by the local structure, in analogy with ref. 21.

We note as an aside that the late decay of *Q*(Δ*t*) is well fitted by the functional form 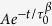 (solid lines), as usually found in glassy materials. The estimated value of the exponent poorly fluctuates around *β* ≃ 0.55 at all the volume fractions investigated, except for the largest one, where it jumps to *β* ≃ 1.75 (see Inset of Fig. 3a). A similar crossover from stretched (*β* < 1) to compressed exponential (*β* > 1) has been previously reported in nearly hard sphere colloidal glasses^23^ as well as in other glassy systems, and its origin is currently debated^43^,^44^.

The decay of *Q*(Δ*t*) allows for estimating the structural relaxation time, *τ*, from the relation *Q*(*τ*) = 1/*e*. Figure 3b shows that the dependence of *τ* on the volume fraction is compatible with a power-law, (Φ − Φ_*c*_)^−*γ*^, as predicted by Mode Coupling Theory (MCT). In particular, we find Φ_*c*_ ≃ 0.80 ± 0.01 and *γ* = 2.5 ± 0.1. The figure also shows the relaxation times of the ISSFs, clarifying that the results of Figs 2b and 3a are fully reflected in the relaxation times. Indeed, *τ* is nearly overlapped to the relaxation time of *F*_*s*_(*k*_2_, Δ*t*) and in between those of *F*_*s*_(*k*_1_, Δ*t*) and *F*_*s*_(*k*_3_, Δ*t*), all the relaxation times being compatible with the same power-law.

The emergence of dynamic heterogeneity can be now characterized defining the dynamic susceptibility from the fluctuations of the DVA dynamic order parameter, *Q*:


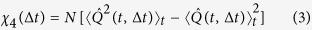


which is directly related to fluctuations of the variance using eq. 1, 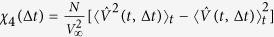.

**Figure 4:**
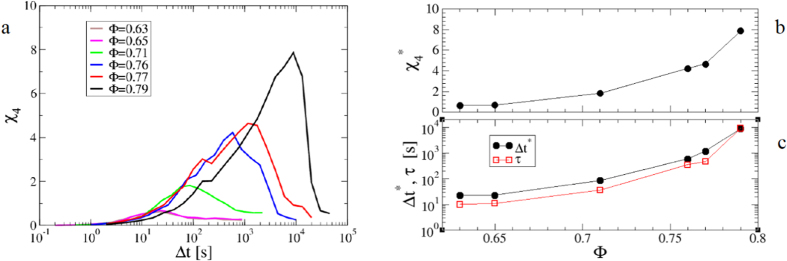
Quantification of Dynamic Heterogeneities. (**a**) Dynamic susceptibility, *χ*_4_, as function of the time-lag Δ*t* for different volume fractions, as indicated. *χ*_4_(Δ*t*) shows a maximum 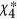 at a time Δt^*^. (**b**) 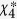 as a function of the volume fraction, Φ. (**c**) Δt^*^ as a function of Φ is compared to the relaxation time, *τ*.

Figure 4a shows that *χ*_4_(*t*) has the typical behaviour reported for the dynamic susceptibility in glass-formers^1^, with a maximum 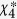 at a time Δt^*^, both clearly increasing on increasing the volume fraction. 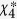 and Δt^*^ roughly estimate the typical size and life-time of clusters of particles dynamically correlated. Accordingly, the mentioned similarities with ordinary critical phenomena emerge since these dynamical clusters become increasingly spatially extended and long-living on approaching the glass transition^1^. Figure 4b and c show that for this system 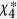 increases about a decade over the investigated range of volume fractions, while Δt^*^ spans almost three orders of magnitude, roughly mimicking the behaviour of the relaxation time, *τ*. Let us stress that DVA allows for a simple and efficient measure of the dynamic susceptibility, since it does exploit the whole statistics provided by the raw video microscopy data and is directly applicable, without preprocessing the images or resolving individual particle positions. Using single particle tracking, instead, we can provide a reliable measurement of the ISSF, but not of the associated susceptibility, 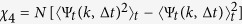. Indeed, the ISSF can be computed averaging over all the tracked particles, no matter the initial and the final time of each trajectory, whereas computing the associated fluctuations, and in particular the square average, 〈Ψ_*t*_(*k*, Δ*t*)^2^〉_*t*_, requires the trajectories to be temporally overlapped during time-windows much larger than the relaxation time. As this time-window increases, more and more trajectories are rejected since particles can diffuse away from the field of view or due to incidental failure of the tracking algorithm. This strongly limits the number of available trajectories, especially at high density, where the relaxation time is large, and large statistics should be required to properly estimate *χ*_4_. For example, at the largest volume fraction, we are able to record only a few tens trajectories that are both temporally overlapped and longer than *τ*.

### Direct visualization of Dynamic Heterogeneities

**Figure 5:**
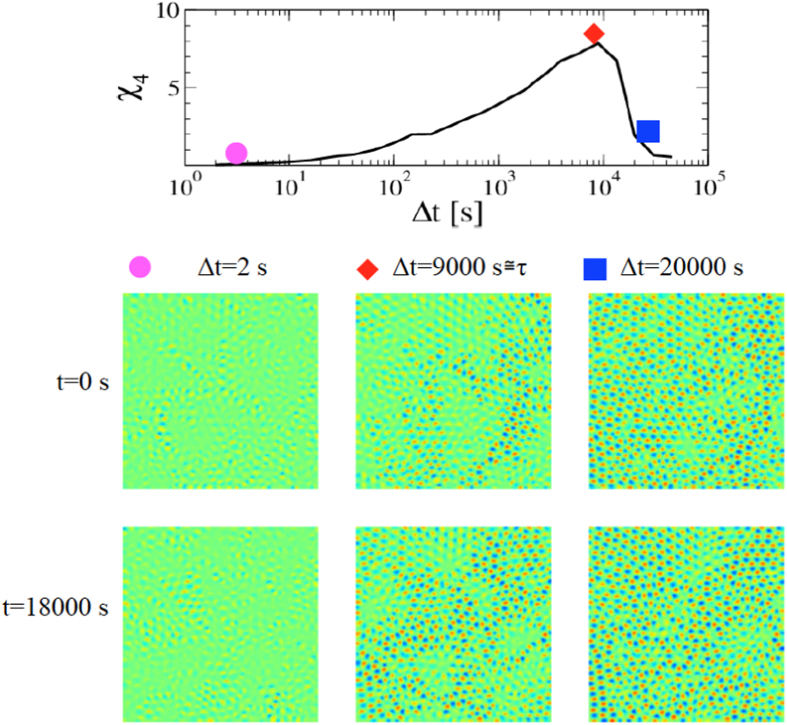
Visualization of Dynamic Heterogeneities. Matrix of differential frames with different initial times *t* and time-lags Δ*t*, for a portion of a sample at the largest investigated volume fraction, Φ = 0.79. For each line, *t* is fixed and Δ*t* increases moving from left to right, as indicated. The first and the third frame of each line report values of Δ*t* much smaller and much larger than the relaxation time, *τ*, while 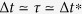 for the second one. The two lines refer to different initial times *t*, separated by a delay of the order of 2*τ*. At Δ*t* ≃ *τ*, dynamic heterogeneities are manifested, either in each single frame as large spatial correlations among the dipoles corresponding to the moved particles, or as fluctuations of the number of dipoles between included in the two lines. In order to have a comparison with quantitative measure of DHs, the upper panel reports *χ*_4_(Δ*t*) for the same volume fraction. The symbols highlight the values of *χ*_4_ at the same time-lags reported in the matrix.

Now we turn back to direct observation of differential frames, in order to show how this approach naturally leads to a novel and effective visualization of DHs. To this aim, Fig. 5 (lower panel), shows a matrix of differential frames at the largest investigated volume fraction, Φ = 0.79. Moving along a line, the initial time, *t*, is fixed, while the time-lag Δ*t* is increasing and the system is progressively relaxing with respect to the initial configuration. Note that the value of Δ*t* corresponding to the central frame is chosen not arbitrarily, but close to the time-lag Δt^*^ of the maximum *χ*_4_, which for this systems is also of the order of the relaxation time, *τ*. The two lines of the matrix correspond to different initial times *t*. Since these *t*′*s* are separated by a time larger than the relaxation time, *τ*, the corresponding configurations are uncorrelated and, therefore, akin to different replicas of the same system, as commonly generated in numerical simulations. For a comparison with quantitative results, the upper panel reports *χ*_4_(Δ*t*) at the same volume fraction, highlighting its value at the time-lags considered in the matrix below. The Figure shows that in each frame at 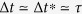 (second column), dipoles corresponding to moved particles exhibit large spatial correlations, and coexist with extended frozen swarms (Δ*I* ≃ 0) where the system has not yet relaxed. The emerging picture resembles the dynamic phase coexistence scenario, which ascribes the glassy dynamics to the temporary coexistence of a mobile/liquid and an immobile/solid phase^45^. Conversely, the differential frames look quite homogeneous at short and long time (first and third columns). Overall, this is reflecting the quantitative informations provided by *χ*_4_(Δ*t*) which is maximum around Δ*t* ≃ *τ*, and small at shorter and longer time (see the upper panel). Furthermore, comparing the two lines of the matrix allows for an alternative visualization of DHs, which become manifested in the fluctuations of dipole patterns between differential frames at the same Δ*t*, but different *t*. Once again, these fluctuations are marked for differential frames at Δ*t* ≃ *τ*: it appears clearly that a much smaller fraction of the system has relaxed in the upper frame compared to the bottom one, despite that the considered time-lag is the same in the two frames. By contrast, fluctuations are negligible at short and long time. In general, these signatures of DHs become less evident at smaller Φ, where the maximum of *χ*_4_ is also smaller.

Observation of differential frames at Δ*t* ≃ *τ* suggests other interesting features of DHs: for example, dynamical clusters of close dipoles resemble a correlated percolation pattern^46^,^47^,^48^,^49^,^50^. In addition, the shape of these clusters looks more compact than that observed at lower volume fraction (see for example the third frame in Fig. 2a, where Δ*t* is also of the order of the relaxation time at that volume fraction), consistently with a string to compact crossover of cooperative rearrangements on approaching the glass transition^51^,^52^.

## Discussion

The results of the previous Section demonstrate the ability to quantitatively monitor the structural relaxation process and the emergence of DHs, starting from the variance of the differential frames. In particular, *(i)* we introduced a dynamic order parameter, *Q*(Δ*t*), that properly describes the relaxation process, as demonstrated by a comparison with the commonly used ISSF, *(ii)* we measured the structural relaxation time, *τ*, from the decay of *Q*(Δ*t*), and finally *(iii)* the dynamic susceptibility *χ*_4_(Δ*t*) from its fluctuations.

Moreover, this method leads to directly visualizing the relaxation process and the emergence of DHs. In particular, Fig. 5 summarizes what is the key of our approach: as the time Δ*t* passes, an increasing number of well defined dipoles appears, which controls the behaviour of the variance, and, therefore, the dynamic order parameter and its fluctuations. Indeed, these dipoles are the signature of particle rearrangements that lead to the structural relaxation and are of order of one particle diameter, since this is the length scale probed by *Q*(*t*). Considering the strongly intermittent nature of particle motion in glasses^3^,^53^, it is likely that variations in the differential frames with Δ*t* are mainly due to the increase of the number of dipoles, rather than to change of their intensity. Accordingly, we have seen that, focusing on dipole patterns, DHs naturally emerge in the differential frames. Incidentally, we mentioned that DHs have a double-sided nature: they are manifested both as spatial correlations in the particle displacements and ensemble fluctuations of the dynamic order parameter. Accordingly, the dynamic susceptibility can be equivalently defined from *(I)* the space integral of *G*_4_(*r*, Δ*t*) or from *(II)* the ensemble fluctuations of the dynamic order parameter, like in eq. 3. However, to measure *χ*_4_ in practice, *(II)* is largely preferred to *(I)*. Experimental techniques, such as DLS, do not give information on the particle positions, but only on the dynamic order parameter and its fluctuations. Even in simulations, where the particle positions can be fully resolved, *(I)* is poorly used in practice, due to the difficulty in obtaining reliable measurements of *G*_4_(*r*, Δ*t*)^1^. Nevertheless, correlations in the particle displacements provide important qualitative insights, being at the base of DH direct visualization proposed until now, for example highlighting the position of the “fast particles”, i.e. the particles which have moved more than a given threshold over a time interval of the order of the relaxation time^3^. We have seen that our approach leads to a similar goal easily, since such an information is already contained in the raw differential frames at Δ*t* ≃ Δt* ≃ *τ*: Particles that have moved significantly give rise to a dipoles and DHs become apparent as spatial correlations among these dipoles. In addition, DHs are also manifested as large fluctuations of the number of dipoles between differential frames at time-lag Δ*t* ≃ Δt* ≃ *τ*, and different initial times, t. This latter is a way to directly visualize the ensemble fluctuations of the dynamic order parameter *(II)*, which are actually used to compute *χ*_4_.

While previous efforts on drying paints used an arbitrary fixed time-lag^54^, we remark the importance of choosing a well defined timescale, namely Δt^*^, to effectively visualize DHs. This timescale is determined by the dynamics and, therefore, changes on varying the system control parameter (Φ in our experiment).

## Conclusions

In this paper, we introduced DVA as a novel and simple experimental method to characterize the dynamics of hard-sphere colloidal glasses of micron-sized particles. We expect DVA to be applicable to a large range of systems formed by different colloidal particles, such as soft particles or attractive particles, which likely form gels, as well as red blood or epithelial cells, and, with three dimensional systems imaged by confocal microscopy. These experimental systems are very popular and a large amount of imaging data have been collected during the last years and mainly analysed by single particle tracking. Previously recorded videos can be easily reprocessed utilizing this approach to obtain information, complementary to that provided by particle tracking and an effective direct visualization of the heterogeneous dynamics. Moreover, preliminary results suggest that DVA could be also suited to systems formed by much smaller (in the nanometer range) primary particles.

Understanding whether the heterogeneous dynamics in glasses had a structural origin is still one of the most relevant open issue in condensed matter physics^2^,^55^. Indeed, the presence of structural heterogeneities implies that of DHs but the opposite is not true^7^. Accordingly, DHs are predicted by several theoretical scenarios both postulating a structural^56^ or a purely dynamic origin^57^ for the glass transition. By contrast, the heterogeneous dynamics of other materials, like gels or fiber networks, is known to have a structural origin. Yet, in practice, structural characterization of these materials requires quite complex experimental efforts, and inferring structural informations from the dynamics can be often an easier alternative. Since these systems are widespread in industry and biology, our method could be very convenient to control their degree of heterogeneity, focussing on the dynamics.

Finally, we suggest that the DVA strategy could be interestingly extended to a wide variety of data sets, not only to video microscopy and imaging data.

## Methods

Data were obtained from previous experiments^39^, which investigated the intermittent single particle motion using particle tracking. We considered quasi-two dimensional hard-sphere-like colloidal systems at different volume fractions, Φ. Precisely, the samples consisted in a 50:50 binary mixture of silica beads dispersed in a water surfactant solution (Triton X-100, 0.2% v/v), to avoid particle sticking through van der Waals forces. Large and small beads had diameters 3.16 and 2.31 *μm*, respectively, resulting in an 1.4 ratio known to prevent crystallization. The systems were imaged using a standard microscope equipped with a 40x objective (OlympusUPLAPO 40XS) and the images were recorded using a fast digital camera (Prosilica GE680). At the highest volume fraction, roughly a thousand particles in the field of view of the microscope were imaged. We focused on a volume fraction range, where the samples can be equilibrated on the experimental timescale and monitored the dynamics after thermal equilibrium is attained. Videos recorded at each volume fraction were several times larger than the relaxation time, *τ*. In particular, video durations and frames per seconds (fps) ranged in [10^3^ *s*, 10^5^ *s*] and [0.5 *s*^−1^, 5 *s*^−^1], respectively, depending on the volume fraction, i.e. larger duration and smaller fps at larger volume fraction. Data analysis was performed using Python and different SciPy libraries^58^. Interactive data exploration and visualization was performed using IPython and Jupyter notebooks^59^. DVA code is freely available on the corresponding author website, http://rpastore.altervista.org.

## Additional Information

**How to cite this article**: Pastore, R. *et al*. Differential Variance Analysis: a direct method to quantify and visualize dynamic heterogeneities. *Sci. Rep.* **7**, 43496; doi: 10.1038/srep43496 (2017).

**Publisher's note:** Springer Nature remains neutral with regard to jurisdictional claims in published maps and institutional affiliations.
